# Continuous Theta Burst Stimulation (cTBS) on Left Cerebellar Hemisphere Affects Mental Rotation Tasks during Music Listening

**DOI:** 10.1371/journal.pone.0064640

**Published:** 2013-05-28

**Authors:** Silvia Picazio, Massimiliano Oliveri, Giacomo Koch, Carlo Caltagirone, Laura Petrosini

**Affiliations:** 1 IRCCS Santa Lucia Foundation, Rome, Italy; 2 Department of Psychology, “Sapienza” University of Rome, Rome, Italy; 3 Department of Neuroscience, “Tor Vergata” University of Rome, Rome, Italy; 4 Department of Psychology, University of Palermo, Palermo, Italy; University of Bologna, Italy

## Abstract

Converging evidence suggests an association between spatial and music domains. A cerebellar role in music-related information processing as well as in spatial-temporal tasks has been documented. Here, we investigated the cerebellar role in the association between spatial and musical domains, by testing performances in embodied (EMR) or abstract (AMR) mental rotation tasks of subjects listening Mozart Sonata K.448, which is reported to improve spatial-temporal reasoning, in the presence or in the absence of continuous theta burst stimulation (cTBS) of the left cerebellar hemisphere. In the absence of cerebellar cTBS, music listening did not influence either MR task, thus not revealing a “Mozart Effect”. Cerebellar cTBS applied before musical listening made subjects faster (*P = *0.005) and less accurate (*P* = 0.005) in performing the EMR but not the AMR task. Thus, cerebellar inhibition by TBS unmasked the effect of musical listening on motor imagery. These data support a coupling between music listening and sensory-motor integration in cerebellar networks for embodied representations.

## Introduction

Revealing the neural bases of music processing has become a central theme in cognitive neuroscience. A peculiar musical phenomenon is the so-called “Mozart Effect”, a short-term enhancement of spatial-temporal reasoning ability following exposure to the Mozart Sonata for Two Pianos in D Major (K.448) [1], [2]. The correlation between spatial and musical domains is supported also by the SMARC effect (Spatial-Musical Association of Response Codes), whereby high-frequency pitches prime “spatially” up responses while low-frequency pitches prime down responses [3], [4]. Mozart’s Sonata listening is reported to evoke activations in DLPFC, occipital cortex and cerebellum, in comparison to Beethoven’s Für Elise or 1930s piano music that evoke activations limited to temporal auditory area [5]. Furthermore, marked activation of cerebellar areas as possible centre controlling motor and perceptual timing [6] and processing melody, harmony and rhythm components of musical task has been described [7], [8]. Experimental and neuroimaging studies document a cerebellar role in spatial functions in general [9], [10], and in mental rotation in particular [11]–[15]. The present study was aimed to investigate the involvement of the cerebellum in the association between spatial and musical domains because this structure appears to play a role in visuo-spatial as well as musical perception. Since in previous studies mental rotation tasks have been used to test for a correlation between spatial and musical abilities [16], [17], in the present research we tested the performances in mental rotation (MR) tasks of healthy adult subjects passively listening Mozart’s Sonata K.448 in the presence or in the absence of continuous theta burst stimulation (cTBS) applied to the left cerebellar hemisphere. Although bilateral cerebellar activations have been observed in both musical [6] and mental rotation tasks [18], to transiently down-regulate the neuronal excitability [19], [20] the cTBS was applied on the left hemisphere, because the activation of left lateral crus I is reported to be associated with the presentation of auditory stimuli [21], [22] and during mental rotation tasks [10], [15]. Since the nature of the stimulus to be rotated in the MR tasks (body *vs.* non-body parts) seems to affect the implicit selection of a particular type of mental transformation (i.e., egocentric or allocentric) [23], [24], in the present study the mental rotation abilities were tested on two different MR tasks: one “embodied” (EMR, Embodied Mental Rotation) requiring to mentally rotate a schematic drawing of the human body from an egocentric point of view, and another “abstract” (AMR, Abstract Mental Rotation) requiring to mentally rotate non-representational figures without any affordance property.

We expected that the down-regulation of the cerebellar activity by cTBS would result in an enhanced Mozart Effect in the hypothesis that by subtracting the cerebellar contribution we would stress the system and allow Mozart Effect to emerge. Music listening could modulate MR performance, either in baseline trials, as predicted by the Mozart’s effect, or by contrasting the disrupting effect of cTBS on the left cerebellum.

## Materials and Methods

### Participants

A sample of 112 neurologically intact subjects [36 males (30.2%); mean age ± SD = 24.7±3.7 years; range 18–35; schooling >13 years] was recruited from Universities and hospital personnel by local advertisement.

Subjects were randomly assigned to one of eight experimental groups. No age [one-way ANOVA: *F*_7,104_ = 0.69, *P* = 0.68] and schooling [one-way ANOVA: *F*_7,104_ = 0.17, *P* = 0.99] differences among groups were found. All participants were right-handed as assessed with the Edinburgh Handedness Inventory [25]. Subjects reported normal- or corrected-to-normal vision and no hearing problems. People with intracranial metallic plates, cardiac pacemakers and with a family or personal history of epilepsy were excluded.

No participant included in the study was musician or even non-professional player of some musical instrument as investigated with a brief telephone interview at the time of recruitment. Furthermore, no included person declared to be a lover or an expert on classical music. The study was approved by the Local Ethics Committee of the IRCCS Santa Lucia Foundation and written consent was obtained from all participants after a full explanation of the procedures of the study.

### Musical Listening

Participants belonging to “Music” groups (cTBS-Music; Sham-Music) were asked to listen to Sonata in D Major K.448 for two pianos and orchestra by Mozart lasting 11 minutes and 28 seconds. Listening was done through Philips stereo earphones. Timing of the MR tasks was designed to be virtually identical to that of musical track. Listening volume was standard for all subjects and set at a high but comfortable level.

### Mental Rotation Tasks

Participants were tested in a quiet room of our lab. They sat comfortably on an armchair at a distance of about 80 cm from a computer monitor; the centre was aligned with the subject’s eyes. Computerized versions of the two different MR tasks were used. Subjects performed MR tasks immediately after the cessation of the cerebellar cTBS.

Before practice phase, participants read on the computer screen a standardized explanation of the task accompanied by visual examples. They were asked to place their right index finger on one of two central keys of a compatible button box located on their legs, which recorded RTs with 1-msec accuracy. Depending on the MR task they were performing, a right button press indicated a “right/same” response, while a left button press indicated a “left/different” response. The practice phase was conducted in silence (without any music listening). To obtain comparable baseline mental rotation performances, all subjects underwent a practice phase for both MR tasks. All participants entered the test phase only when they reached a minimum of 80% of correct responses in the practice phase. The number of trials needed to reach the criterion did not statistically differ among groups [*F*_7,104 = _0.26, *P* = 0.97].

**Figure 1:**
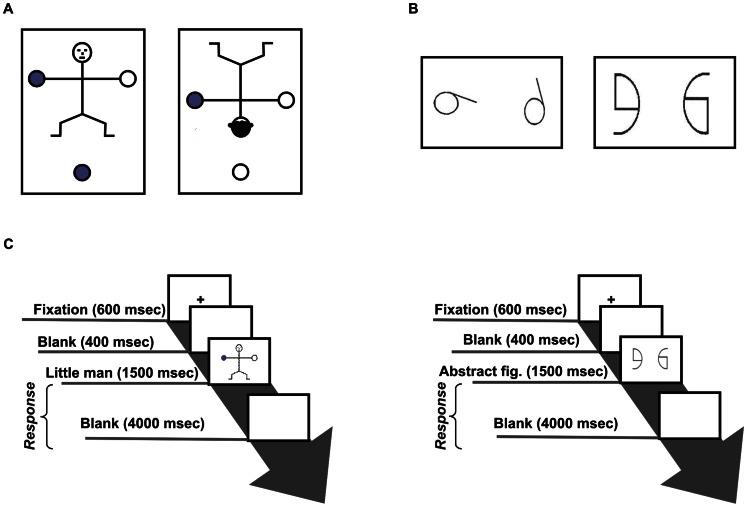
Stimuli and timing. Two examples of EMR items (A). Frontal and back views at rotation angles of 0° and 180° are illustrated. In this figure the red dot actually shown to the participants is depicted as white and the blue one as grey. In the first item the correct response is “right”, while in the second one it is “left”. Two examples of AMR items (B). In the first item the correct response is “different”, while in the second one it is “same”. Timing of EMR and AMR tasks (C).

*Embodied Mental Rotation* (EMR) task is a modified version of the Ratcliff’s Little Man [26] programmed to run on computer. The Ratcliff’s Little Man test presents images of a schematic drawing of a human body seen from different orientations in an equal number of trials: 0°, 90° (to the right and to the left), 180° angles either in frontal or back views. EMR task requires identifying which hand (right or left) was marked in red or blue depending on the stimulus dot presented in the lower portion of the figure similar to the “which hand task” adopted by Zacks et al. [27] (Fig. 1A). The colour dot variable was added to prevent response memorization and increase the number of trials. The number of right and left dots was balanced across trials. The EMR stimuli differed from the originals of the Ratcliff’s Little Man [26] for number of trials (128 *vs.* 32), colours (red and blue *vs.* black and white) and for the presence of a dot visual reference below the figure.

For the practice phase we selected 8 pictures (balanced as above described). Each figure was presented 4 times in a randomized order, for a total of 32 trials.

In the test phase, we used all the 32 images, 8 of whom were also presented in the practice phase. Each of the 32 images was repeated 4 times, for a total of 128 trials presented in a randomized order. The task was programmed and run by using Psyscope for Macintosh. In both practice and test phases, before the presentation of each image, subjects had to fixate a central black cross remaining on the blank screen for 600 ms. Following the fixation cross disappearance, there was a blank interval of 400 ms. After this interval, the image of the little man was presented for 1500 ms. Following stimulus disappearance, a blank screen was again displayed for a maximum of 4000 ms or until key pressing. Then, the fixation cross reappeared and the next trial began. Subjects answered “right” or “left” according to which little man’s hand they retained to be marked by the stimulus dot. Only responses given during image presentation or during the subsequent 4000 ms (blank screen) were considered as valid and recorded (Fig. 1C).

Parameters considered were: *Reaction times (RTs)*, i.e. the time interval between image presentation and key pressing; *Accuracy*, i.e. number of correct responses. *Angular disparity index*, i.e. the RTs of the trials when the little man was presented in its prototypical orientation (0°) or rotated away from it (90° to the right, 90° to the left, 180°) and looked in frontal or back views.

*Abstract Mental Rotation* (AMR) task is a modified version of Thurstone’s primary mental ability test cards [28] programmed to run on computer. The task required to provide “same/different” responses at the presentation of pairs of similar two-dimensional abstract figures differently rotated from each other. The pairs of images were made up of four different abstract figures (Fig. 1B). Subjects had to respond “same” if they retained that the two images were overlapping by performing an operation of mental rotation on the plan, or “different” if the two images were mirrored. Figures were balanced so that each pair of images was displayed exactly one quarter of the total times and the correct answer was “same” or “different” in an equal number of trials.

For the practice phase of this task, we selected 8 pairs of the four figures; each of them was presented 4 times in a randomized order for a total of 32 trials.

For the test phase, we used all the 98 images with the repetition of some of them to reach a total of 128 trials presented in a randomized order. AMR test was programmed and played by using Psyscope on a Macintosh computer. Times, method and parameters were the same as described for the EMR task, with the only difference that in AMR task participants provided the answer “same” or “different” rather than “right” or “left” (Fig. 1C).

### TMS Procedure

A MagStim Super Rapid magnetic stimulator (Magstim Company, Whitland, Wales, UK), connected with a figure-of-eight coil with a diameter of 90 mm was used to deliver TBS over the scalp site corresponding to the left lateral cerebellum. The magnetic stimulus had a biphasic waveform with a pulse width of about 300 µs. Three-pulse bursts at 50 Hz repeated every 200 ms for 40 s (equivalent to “continuous theta burst stimulation” cTBS) were delivered at 80% of the Active Motor Threshold (AMT) over left lateral cerebellum (600 pulses). AMT was tested over the motor cortex of the right hemisphere. AMT was defined as the lowest intensity that produced MEPs of >200 µV in at least five out of 10 trials when the subject made a 10% of maximum contraction using visual feedback [29]. The inhibitory effect of cTBS with these characteristics is supposed to last about 60 min [19]. TMS was applied over the left lateral cerebellum using the same scalp coordinates (1 cm inferior and 3 cm left to the inion) adopted in previous studies, in which MRI reconstruction and neuro-navigation systems showed that cerebellar TMS in this site predominantly target the posterior and superior lobules of the lateral cerebellum [30], [31]. Although cerebellar stimulation has been originally performed with a double cone coil 31 we used the figure-of-eight coil, since this approach has been adopted in previous investigations in which cerebellar rTMS was shown to be effective in modulating the excitability of the contralateral motor cortex [32], [33]. The coil was positioned tangentially to the scalp, with the handle pointing superiorly. This orientation is able to modulate contralateral M1 excitability [32] and to interfere with cognitive functions such as procedural learning and sub-second time perception when a 1 Hz rTMS paradigm is adopted [32], [34], [35]. The exact coil position was marked by an inking pen to ensure an accurate positioning of the coil throughout the experiment. The stimulating coil was held by hand and coil position was continuously monitored throughout the experiment. For sham cTBS the coil was positioned over the same scalp site, but angled away so that no current was induced in the brain.

### Procedure

#### Experiment 1 (EMR task)

Fifty-six subjects [16 males (28.5%)] were randomly assigned to one of four experimental groups, with 14 subjects in each group: cTBS-Music group, in which the subjects performed the EMR task during Mozart’s Sonata listening following cTBS of the left cerebellar hemisphere; cTBS-Silence group, in which the subjects performed the EMR task in silence following cTBS of the left cerebellar hemisphere; Sham-Music group, in which the subjects performed the EMR task during Mozart’s Sonata listening following sham cTBS of the left cerebellar hemisphere; Sham-Silence group, in which the subjects performed the EMR task in silence following sham cTBS of the left cerebellar hemisphere.

#### Experiment 2 (AMR task)

Fifty-six subjects [20 males (35.7%)] were randomly assigned to one of four experimental groups, with 14 subjects in each group: cTBS-Music group, in which the subjects performed the AMR task during Mozart’s Sonata listening following cTBS over the left cerebellar hemisphere; cTBS-Silence group, in which the subjects performed the AMR task in silence following cTBS over the left cerebellar hemisphere; Sham-Music group, in which the subjects performed the AMR task during Mozart’s Sonata listening following sham cTBS over the left cerebellar hemisphere; Sham-Silence group, in which the subjects performed the AMR task in silence following sham cTBS over the left cerebellar hemisphere.

To assess any interference between EMR and AMR tasks, in a pilot study we submitted a sample of 6 subjects to both MR tasks. We noted that the execution of the first task (whatever it was) biased the execution of the second one as for the “strategy” (egocentric or allocentric) used to solve the task. Furthermore, another sample of 6 subjects was submitted twice to the EMR task. RTs of the two executions significantly differed [*F*_1,5 = _7.39, *P* = 0.04]. Although the accuracy scores of two executions did not reach a significant difference [*F*_1,5 = _5.0, *P* = 0.07], the tendency toward a practice effect was confirmed. Thus, a between-subject experimental design was adopted.

### Statistical Analyses

Two four-way ANOVAs with Stimulation (cTBS *vs.* Sham), Listening (Music *vs.* Silence), Task (EMR *vs.* AMR) and Gender (males *vs.* females) as between-subject factors were performed separately on RTs and accuracy scores.

In Experiment 1, a five-way ANOVA on RTs recorded according to the different orientations of the little man (Angular Disparity Index) with Stimulation (cTBS *vs.* Sham), Listening (Music *vs.* Silence) and Gender (males *vs.* females) as between-subject factors and View (frontal *vs.* back) and Angle (0°, 90° to the right, 90° to the left, 180°) as within-subject factors was calculated. A three-way ANOVA on EMR accuracy scores with Stimulation (cTBS *vs*. Sham), Listening (Music *vs.* Silence) and Gender (males *vs.* females) as between-subjects factors was carried out.

In Experiment 2, two three-way ANOVAs with Stimulation (cTBS *vs*. Sham), Listening (Music *vs.* Silence) and Gender (males *vs.* females) as between-subjects factors were performed separately on RTs and accuracy scores. Furthermore, one-way ANOVAs on RTs (or accuracy scores) of several groups were calculated. *Post hoc* Tukey’s tests were performed when required. The threshold of significance was set at *P*<0.05.

## Results

RTs for correct responses are presented, although analogous results were obtained when correct and incorrect responses were measured.

Four-way ANOVA (Stimulation × Listening × Task × Gender) on RTs revealed a significant Task effect [*F*_1,96 = _61.84, *P*<0.001] as well as a significant interaction [Stimulation × Listening × Task: *F*_1,96 = _6.39, *P* = 0.013]. Stimulation [*F*_1,96 = _0.07, *P* = 0.79] and Gender [*F*_1,96 = _1.14, *P* = 0.29] factors were not significant. Listening factor was close to significance [*F*_1,96 = _3.47, *P* = 0.065]. Similarly, four-way ANOVA (Stimulation × Listening × Task × Gender) on accuracy scores revealed a significant Task [*F*_1,96 = _3.44, *P*<0.066] effect as well as a tendency for a first-order interaction: [Stimulation × Listening: *F*_1,96 = _3.93, *P* = 0.065]. Stimulation [*F*_1,96 = _1.25, *P* = 0.265], Listening [*F*_1,96 = _3.09, *P* = 0.082] and Gender [*F*_1,96 = _0.91, *P* = 0.342] factors were not significant. Since the two tasks were significantly different, all subsequent analyses were performed separately for each MR task.

### Experiment 1 (EMR task): RTs According to the Angular Disparity Index

**Figure 2:**
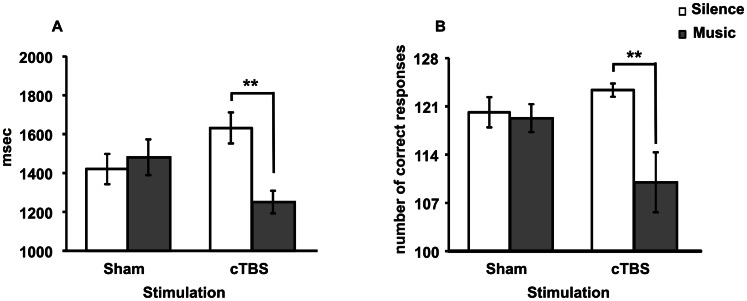
RTs (A) and accuracy (B) of EMR task. No significant difference was found between Sham-Silence and Sham-Music groups. cTBS-Music group was significantly faster and less accurate than cTBS-Silence group. The asterisks indicate significance level: ** *P*<0.01. In this and in the following figures, graph bars represent standard errors.

**Figure 3:**
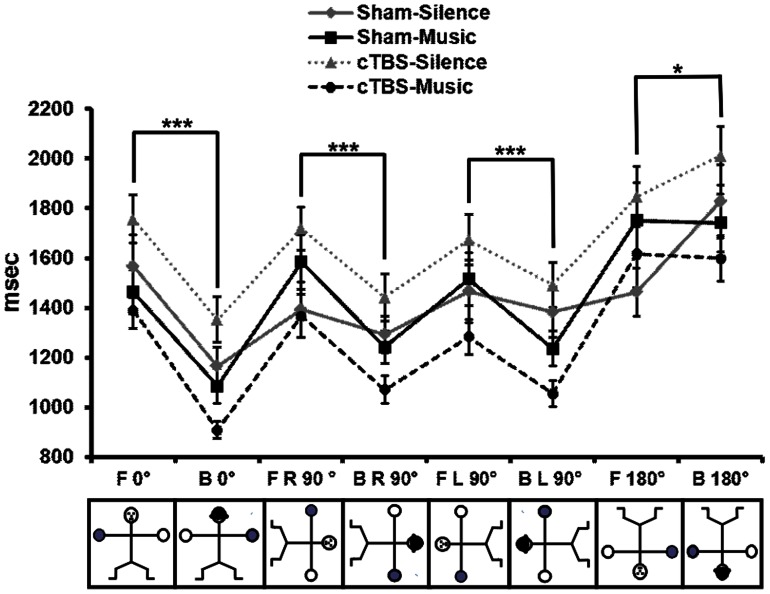
Angular disparity of EMR task. RTs in the four experimental groups according to angles [0°, 90° to the right (R), 90° to the left (L), 180°] and views [frontal (F), back (B)] of the stimulus. The asterisks indicate significance level: * *P*<0.05; *** *P*<0.001.

A five-way ANOVA (Stimulation × Listening × Gender × View × Angle) on RTs according to the Angular Disparity Inde*x* revealed significant Listening [*F*_1,48_ = 7.70, *P* = 0.007], View [*F*_1,48_ = 51.18, *P*<0.001] and Angle [*F*_3,144_ = 37.65, *P*<0.001] factors, while Stimulation [*F*_1,48_ = 0.21, *P* = 0.642] and Gender [*F*_1,48_ = 0.11, *P* = 0.219] factors were not significant. First-order interactions [Stimulation × Listening: *F*_1, 48 = _4.11, *P* = 0.048; Listening × Angle: *F*_3,144_ = 2.71, *P* = 0.047; View × Angle: *F*_3,144_ = 35.94, *P*<0.001] were also significant. *Post hoc* comparisons on Stimulation × Listening interaction indicated that in the presence of cTBS the participants were significantly (*P* = 0.002) faster when listening to music with respect to the silence condition. The two Sham conditions did not differ in the presence or in the absence of music listening (*P* = 0.93) (Fig. 2A; Fig. 3).

### Experiment 1 (EMR task): Accuracy Scores

The percentages of incorrect trials were 14% in cTBS-Music, 4% in cTBS-Silence, 7% in Sham-Music, 6% in Sham-Silence groups. A three-way ANOVA (Stimulation × Listening × Gender) on accuracy scores revealed a significant Listening [*F*_1,48_ = 4.40, *P* = 0.041] effect as well as a significant first-order interaction [Stimulation × Listening: *F*_1,52_ = 4.14, *P* = 0.047]. Stimulation [*F*_1,48_ = 0.54, *P* = 0.464] and Gender [*F*_1,48_ = 0.67, *P* = 0.418] factors were not significant. *Post hoc* comparisons on the interaction indicated that in the presence of cTBS the participants were significantly (*P* = 0.010) less accurate when listening to music with respect to the silence condition. The two Sham conditions did not differ in the presence or in the absence of music listening (*P* = 0.999) (Fig. 2B).

These findings indicate that performances in the EMR task were modulated by cTBS of the left cerebellar hemisphere only during music listening. Namely, during Mozart’s Sonata listening, subjects submitted to cTBS displayed performances faster but less accurate.

### Experiment 2 (AMR Test): RTs

**Figure 4:**
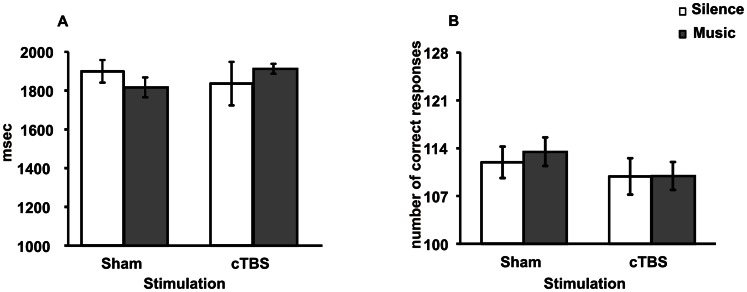
RTs (A) and accuracy (B) of AMR task. No significant difference was found among groups in any condition.

A three-way ANOVA (Stimulation × Listening × Gender) on RTs failed to reveal significant Stimulation [*F*_1,48_ = 0.18, *P = *0.675] Listening [*F*_1,48_ = 0.18, *P = *0. 675] and Gender [*F*_1,48_ = 0.02, *P = *0.888] effects. No significant interactions were found (Fig. 4A).

### Experiment 2 (AMR Test): Accuracy Scores

The percentages of incorrect trials were 14% in cTBS-Music, 14% in cTBS-Silence, 11% Sham-Music, 12% in Sham-Silence groups. A three-way ANOVA (Stimulation × Listening × Gender) on accuracy scores failed to reveal significant Stimulation [*F*_1,48_ = 0.75, *P* = 0.390] Listening [*F*_1,48_ = 0.02, *P* = 0.890] and Gender [*F*_1,48_ = 0.24, *P* = 0.623] effects. No significant interactions were found (Fig. 4B).

These findings indicate that performances in the AMR task were not modulated by cTBS over the left cerebellar hemisphere, or by music listening in terms of velocity and accuracy.

## Discussion

The aim of the present study was to analyze the role of cerebellar networks when spatial and musical contents interact. In particular, we studied whether and how the down-regulation of the neuronal excitability of the left cerebellar hemisphere interfered with two mental rotation tasks, one embodied and one abstract, performed during the listening to Mozart’s Sonata K.448. In other words, we tested for a Mozart Effect on MR tasks during cerebellar inhibition.

The presence of the music did not result in differences in both mental rotation tasks in the absence of cerebellar inhibition (Sham condition), excluding thus the occurrence of the Mozart Effect. However, a modulatory effect of Mozart’s Sonata listening on embodied mental rotation performance emerged following the down-regulation of the left cerebellar hemisphere neuronal activity. Namely, music listening rendered subjects faster and less accurate in performing the EMR task following cerebellar cTBS. Such a finding outlines a speed-accuracy trade-off that fully fits the trading relationship between speed and accuracy repeatedly described in studies on decision-making performances [36]–[39].

The combined effect of inhibitory stimulation and music listening could be explained by the state-dependency theory advancing that TMS behavioral effects are determined by the initial activation state of the stimulated structure [40], [41]. Although in a speculative way, we advance the hypothesis that the state-dependency of TMS effects could also work in the reverse direction: cerebellar pre-conditioning through cTBS could have primed neuronal populations either at the level of the stimulated cerebellum and/or of the contralateral cerebral hemisphere, facilitating the effects of subsequent music listening on embodied mental rotation task. According to this hypothesis, the down-regulation of cerebellar excitability would unmask facilitatory effects of music listening on RTs in the embodied mental rotation task. In this respect, it is worth noting that either cTBS or music listening failed to affect RTs and accuracy scores in the mental rotation tasks when delivered alone.

Interestingly, the effects of combined cerebellar stimulation and music listening were present only when EMR task was performed and fully absent when AMR task was performed.

EMR and AMR tasks appear to be totally different. In performing the abstract task longer RTs and smaller number of correct answers were displayed in respect to the EMR task, a difference probably linked to the two images to be rotated and to the different mental rotation strategies in AMR task.

In EMR the participants decided which was the left or right hand in the pictures of rotated dummies. As previously described [42], most participants tend to solve tasks of rotation of body pictures (EMR) by imaging to move their own bodies from their actual posture into that of the presented stimulus (egocentric strategy). Conversely, when abstract figures have to be rotated (AMR), usually participants tend to solve the task by imaging the “objects” shifted by inanimate forces (allocentric strategy). Actually, AMR stimuli were abstract figures without any affordance property, so the subjects could not imagine grasping or manipulating the object as though it was a tool. Although there are evidences that motor processes may be used in the mental rotation of both body parts and abstract figures [43]–[45], in the absence of explicit requests of using a particular mental rotation strategy, stimuli as body images tend to elicit an egocentric strategy, while abstract figures an allocentric one [46]. Neurophysiologically, egocentric rotation causes a direct mapping into one’s own body schema and involves overall motor processes, while allocentric rotation relies much less on motor processes [47], [48]. The notion that the motor processes are differently involved according to the kind of stimulus and strategy used is supported by several reports. Single pulse TMS applied to the motor area slowed down RTs of participants that mentally rotated hand but not letter stimuli [23], [49]. Patients with sensorimotor cortical lesion or with cervical dystonia showed selective deficit in simulating body part movements compared with object movements [50], [51]. Thus, it appears likely that the EMR task required an involvement of motor system in general, and of cerebellar networks in particular, greater than the AMR task. In keeping with this advance, participants exhibited longer RTs for stimulus orientations (180°) in which actual movements would be more difficult to be performed as emerged by the Angular Disparity Index. Furthermore, RTs were generally shorter in rotating figures in back than in frontal view, suggesting that the duration of mental rotation was influenced by the complexity of the movements to be mentally executed and by proprioceptive information regarding body position [42]. This angle-view interdependence suggests that participants did use a motor strategy to solve EMR task, and further indicates that subjects identified themselves in the little man in providing the response (egocentric strategy) [52].

Therefore, the unexpected finding that combined effects of cTBS and music listening were selective for the EMR task could be explained by the existence of a coupling between music perception and motor activity in cerebellar networks. Notably, a marked cerebellar activation has been described during mental rotation or spatial transformation tasks [10]–[12], [15], [50] and the cerebellum has been implicated in the music perception, namely in the rhythmic sequences [53].

Many examples of sound-movement interactions can be found in our life such as dancing, singing or playing an instrument, as well as the impulse to tap the beat while listening to cadenced music. An increased activation in motor cortex and cerebellar hemispheres during passive listening was also reported [54]. Furthermore, music perception-action mechanisms have been demonstrated in specific populations of dancers [55], [56] and musicians [57], [58] and also in non-musicians [59]. Increasing evidence indicates functional interactions of auditory and motor systems leading to a remarkable sensorimotor interplay [57], [60]–[62]. Motor regions, as the premotor cortex, supplementary motor area, and cerebellum, resonate in response to sounds that do not bear any obvious significance for action implementation, emphasizing an intrinsic coupling between perception-action processes whereby the motor system is sensitive to and driven by properties of the auditory stimulus [61]. The implication is that whenever we hear music, our brain is primed for action. The close intertwining of music and movement, even when not overtly performed, implies that there cannot be a merely passive listening condition. Listening to music involves tracking sequential events over time and this may be of relevance and/or inherent to the motor system.

Speaking of sequential events obviously evokes the cerebellar function of sequencing [66]. Increasing evidence underlines that cerebellar networks integrate sensory and motor information to generate internal models for predictive motor control [63], [64]. An fMRI study in ballet dancers watching dance movements showed increased activity of cerebellar areas [65]. Furthermore, greater cerebellar volume but not total brain volume was found in musicians compared to non-musicians [66]. Musicians’ and dancers’ performances require control motor functions such as timing, sequencing and spatial organization of movement. Interestingly, all of these are functions associated with cerebellar activity [67], [68]. During music listening the cerebellum appears to be engaged to enable temporally controlled movements and optimize the motor outcome. Furthermore, the existence of overlapping neural networks for music processing and movement is suggested by a recent study examining patients affected by Huntington’s disease. The more severe their voluntary and involuntary movement dysfunction, the more increased their cerebellar activation during music processing [69].

Although it was not one of the main objectives of the present study, we did not find any advantage in male participants as often described in literature [70], [71]. It is possible that gender differences did not emerge because of the use of computerized task [72] or more likely because of the high schooling of the sample studied. Previous studies redefined gender differences in mental rotation tasks in terms of comparable behavioral performances [73] and similar cortical activations [74]. Although the sample of the present research was not balanced by gender, no gender differences were found, analogously to other researches using EMR and AMR tasks [72,74].

A limitation of the present study is the lack of a control site to check for a spread of activation towards the ipsilateral occipital cortex that actually cannot be fully excluded. Indeed, the occipital cortex is an essential part of the mental rotation network and of mental (visuo-spatial) imagery processed. However, if so, we would expect a modulation of both MR tasks following occipital TBS, and not of only one. Another limit of the study could be the use of a between-subject design that although avoided any practice effect did not allow testing subjects on two different tasks.

In conclusion, the present results do not support the direct influence of music on visuo-spatial abilities but emphasize the complex effect of music listening on the representation of the human body, emerging only when the excitability of the left cerebellum was down-regulated. These findings suggest that musical-motor synchrony and timing associated with the activity of cerebellar networks as hub of faceted sensory–motor information modulates embodied spatial cognition.

## References

[1] Rauscher FH, Shaw GL, Ky KN (1993) Music and spatial task performance. Nature 365: 611. 10.1038/365611a0 8413624

[2] Rauscher FH, Shaw GL, Ky KN (1995) Listening to Mozart enhances spatial-temporal reasoning: towards a neurophysiological basis. Neuroscience letters 185: 44–47. 7731551 10.1016/0304-3940(94)11221-4

[3] Rusconi E, Kwan B, Giordano BL, Umilta C, Butterworth B (2006) Spatial representation of pitch height: the SMARC effect. Cognition 99: 113–129. 15925355 10.1016/j.cognition.2005.01.004

[4] Lidji P, Kolinsky R, Lochy A, Morais J (2007) Spatial associations for musical stimuli: a piano in the head? J Exp Psychol Hum Percept Perform 33: 1189–1207. 17924817 10.1037/0096-1523.33.5.1189

[5] Bodner M, Muftuler LT, Nalcioglu O, Shaw GL (2001) fMRI study relevant to the Mozart effect: brain areas involved in spatial-temporal reasoning. Neurol Res 23: 683–690. 11680506 10.1179/016164101101199108

[6] Molinari M, Leggio MG, Thaut MH (2007) The cerebellum and neural networks for rhythmic sensorimotor synchronization in the human brain. Cerebellum 6: 18–23. 17366263 10.1080/14734220601142886

[7] Griffiths TD, Johnsrude I, Dean JL, Green GG (1999) A common neural substrate for the analysis of pitch and duration pattern in segmented sound? Neuroreport 10: 3825–3830. 10716217 10.1097/00001756-199912160-00019

[8] Peretz I, Zatorre RJ (2005) Brain organization for music processing. Annu Rev Psychol 56: 89–114. 15709930 10.1146/annurev.psych.56.091103.070225

[9] Molinari M, Petrosini L, Misciagna S, Leggio MG (2004) Visuospatial abilities in cerebellar disorders. Journal of neurology, neurosurgery, and psychiatry 75: 235–240. PMC1738892 14742596

[10] Stoodley CJ, Valera EM, Schmahmann JD (2010) An fMRI study of intra-individual functional topography in the human cerebellum. Behavioural neurology 23: 65–79. 20714062 10.3233/BEN-2010-0268 PMC3776583

[11] Molinari M, Leggio MG (2007) Cerebellar information processing and visuospatial functions. Cerebellum 6: 214–220. 17786817 10.1080/14734220701230870

[12] Vingerhoets G, de Lange FP, Vandemaele P, Deblaere K, Achten E (2002) Motor imagery in mental rotation: an fMRI study. Neuroimage 17: 1623–1633. 12414300 10.1006/nimg.2002.1290

[13] Creem-Regehr SH, Neil JA, Yeh HJ (2007) Neural correlates of two imagined egocentric transformations. Neuroimage 35: 916–927. 17275336 10.1016/j.neuroimage.2006.11.057

[14] Zacks JM (2008) Neuroimaging studies of mental rotation: a meta-analysis and review. J Cogn Neurosci 20: 1–19. 17919082 10.1162/jocn.2008.20013

[15] Stoodley CJ, Valera EM, Schmahmann JD (2012) Functional topography of the cerebellum for motor and cognitive tasks: an fMRI study. Neuroimage 59: 1560–1570. 21907811 10.1016/j.neuroimage.2011.08.065 PMC3230671

[16] Douglas KM, Bilkey DK (2007) Amusia is associated with deficits in spatial processing. Nat Neurosci 10: 915–921. 17589505 10.1038/nn1925

[17] Williamson VJ, Cocchini G, Stewart L (2011) The relationship between pitch and space in congenital amusia. Brain Cogn. 76: 70–6. 10.1016/j.bandc.2011.02.016 21440971

[18] Weiss MM, Wolbers T, Peller M, Witt K, Marshall L, et al (2009) Rotated alphanumeric characters do not automatically activate frontoparietal areas subserving mental rotation. Neuroimage 44: 1063–73. 18977449 10.1016/j.neuroimage.2008.09.042

[19] Huang YZ, Edwards MJ, Rounis E, Bhatia KP, Rothwell JC (2005) Theta burst stimulation of the human motor cortex. Neuron 45: 201–206. 15664172 10.1016/j.neuron.2004.12.033

[20] Koch G, Mori F, Marconi B, Codeca C, Pecchioli C, et al (2008) Changes in intracortical circuits of the human motor cortex following theta burst stimulation of the lateral cerebellum. Clinical Neurophysiology 119: 2559–2569. 18824403 10.1016/j.clinph.2008.08.008

[21] Alluri V, Toiviainen P, Jaaskelainen IP, Glerean E, Sams M, et al (2011) Large-scale brain networks emerge from dynamic processing of musical timbre, key and rhythm. Neuroimage 59: 3677–3689. 22116038 10.1016/j.neuroimage.2011.11.019

[22] Petacchi A, Laird AR, Fox PT, Bower JM (2005) Cerebellum and auditory function: an ALE meta-analysis of functional neuroimaging studies. Hum Brain Mapp. 25: 118–128. 10.1002/hbm.20137 PMC6871682 15846816

[23] Tomasino B, Borroni P, Isaja A, Rumiati RI (2005) The role of the primary motor cortex in mental rotation: a TMS study. Cognitive Neuropsychology 22: 348–363. 21038255 10.1080/02643290442000185

[24] Kosslyn SM, DiGirolamo GJ, Thompson WL, Alpert NM (1998) Mental rotation of objects versus hands: neural mechanisms revealed by positron emission tomography. Psychophysiology 2: 151–61. 9529941

[25] Oldfield RC (1971) The assessment and analysis of handedness: the Edinburgh inventory. Neuropsychologia 9: 97–113. 5146491 10.1016/0028-3932(71)90067-4

[26] Ratcliff G (1979) Spatial thought, mental rotation and the right cerebral hemisphere. Neuropsychologia 17: 49–54. 431809 10.1016/0028-3932(79)90021-6

[27] Zacks J, Rypma B, Gabrieli JD, Tversky B, Glover GH (1999) Imagined transformations of bodies: an fMRI investigation. Neuropsychologia 9: 1029–40. 10.1016/s0028-3932(99)00012-3 10468366

[28] Thurstone LL (1937) Psychology as a Quantitative Rational Science. Science 85: 227–232. 17841375 10.1126/science.85.2201.227

[29] Rothwell JC (1997) Techniques and mechanisms of action of transcranial stimulation of the human motor cortex. Journal of Neuroscience Methods 74: 113–122. 9219881 10.1016/s0165-0270(97)02242-5

[30] Del Olmo MF, Cheeran B, Koch G, Rothwell JC (2007) Role of the cerebellum in externally paced rhythmic finger movements. J Neurophysiol 98: 145–152. 17460103 10.1152/jn.01088.2006

[31] Koch G, Oliveri M, Torriero S, Salerno S, Lo Gerfo E, et al (2007) Repetitive TMS of cerebellum interferes with millisecond time processing. Experimental Brain Research 179: 291–299. 17146647 10.1007/s00221-006-0791-1

[32] Oliveri M, Koch G, Torriero S, Caltagirone C (2005) Increased facilitation of the primary motor cortex following 1 Hz repetitive transcranial magnetic stimulation of the contralateral cerebellum in normal humans. Neuroscience Letters 376: 188–193. 15721219 10.1016/j.neulet.2004.11.053

[33] Fierro B, Giglia G, Palermo A, Pecoraro C, Scalia S, et al (2007) Modulatory effects of 1 Hz rTMS over the cerebellum on motor cortex excitability. Experimental Brain Rsearch 176: 440–447. 10.1007/s00221-006-0628-y 16917771

[34] Torriero S, Oliveri M, Koch G, Caltagirone C, Petrosini L (2004) Interference of left and right cerebellar rTMS with procedural learning. J Cogn Neurosci 16: 1605–1611. 15601522 10.1162/0898929042568488

[35] Torriero S, Oliveri M, Koch G, Lo Gerfo E, Salerno S, et al (2007) Cortical networks of procedural learning: evidence from cerebellar damage. Neuropsychologia 45: 1208–1214. 17166525 10.1016/j.neuropsychologia.2006.10.007

[36] Ivanoff J, Branning P, Marois R (2008) fMRI evidence for a dual process account of the speed-accuracy tradeoff in decision-making. PLoS One 3: e2635. 18612380 10.1371/journal.pone.0002635 PMC2440815

[37] Trimmer PC, Houston AI, Marshall JA, Bogacz R, Paul ES, et al (2008) Mammalian choices: combining fast-but-inaccurate and slow-but-accurate decision-making systems. Proceedings Biological sciences/The Royal Society 275: 2353–2361. 10.1098/rspb.2008.0417 PMC2603220 18611852

[38] Vallesi A, McIntosh AR, Crescentini C, Stuss DT. 2012. fMRI investigation of speed-accuracy strategy switching. Hum Brain Mapp. 33: 1677–1688. 10.1002/hbm.21312 PMC6870457 21618664

[39] Marshall JA, Bogacz R, Gilchrist ID (2012) Consistent implementation of decisions in the brain. PLoS One 7: e43443. 22984425 10.1371/journal.pone.0043443 PMC3440404

[40] Silvanto J, Muggleton N, Walsh V (2008) State-dependency in brain stimulation studies of perception and cognition. Trends in Cognitive Sciences 12: 447–454. 18951833 10.1016/j.tics.2008.09.004

[41] Silvanto J, Pascual-Leone A (2008) State-dependency of transcranial magnetic stimulation. Brain Topography 21: 1–10. 18791818 10.1007/s10548-008-0067-0 PMC3049188

[42] Parsons LM (1994) Temporal and kinematic properties of motor behavior reflected in mentally simulated action. J Exp Psychol Hum Percept Perform 20: 709–30. 8083630 10.1037//0096-1523.20.4.709

[43] Wexler M, Kosslyn SM, Berthoz A (1998) Motor processes in mental rotation. Cognition 68: 77–94. 9775517 10.1016/s0010-0277(98)00032-8

[44] Wohlschlager A, Wohlschlager A (1998) Mental and manual rotation. J Exp Psychol Hum Percept Perform 24: 397–412. 9606108 10.1037//0096-1523.24.2.397

[45] Janczyk M, Pfister R, Crognale MA, Kunde W (2012) Effective rotations: Action effects determine the interplay of mental and manual rotations. Journal of Experimental Psychology General 141: 489–501. 46. Wraga M, Thompson WL, Alpert NM, Kosslyn SM. (2003) Implicit transfer of motor strategies in mental rotation. Brain Cogn 52: 135–43. 10.1037/a0026997 22268853

[46] Kosslyn SM, Thompson WL, Wraga M, Alpert NM (2001) Imagining rotation by endogenous versus exogenous forces: distinct neural mechanisms. Neuroreport 12: 2519–2525. 11496141 10.1097/00001756-200108080-00046

[47] Ruby P, Decety J (2001) Effect of subjective perspective taking during simulation of action: a PET investigation of agency. Nat Neurosci 4: 546–550. 11319565 10.1038/87510

[48] Ganis G, Keenan JP, Kosslyn SM, Pascual-Leone A (2000) Transcranial magnetic stimulation of primary motor cortex affects mental rotation. Cerebral Cortex 10: 175–180. 10667985 10.1093/cercor/10.2.175

[49] Tomasino B, Skrap M, Rumiati RI (2011) Causal role of the sensorimotor cortex in action simulation: neuropsychological evidence. J Cogn Neurosci 23: 2068–2078. 20849231 10.1162/jocn.2010.21577

[50] Fiorio M, Tinazzi M, Ionta S, Fiaschi A, Moretto G, et al (2007) Mental rotation of body parts and non-corporeal objects in patients with idiopathic cervical dystonia. Neuropsychologia 45: 2346–54. 17412373 10.1016/j.neuropsychologia.2007.02.005

[51] Ionta S, Perruchoud D, Draganski B, Blanke O (2012) Body context and posture affect mental imagery of hands. PLoS One 7: e34382. 22479618 10.1371/journal.pone.0034382 PMC3316677

[52] Chen JL, Penhune VB, Zatorre RJ (2005) Tapping in synchrony to auditory rhythms: effect of temporal structure on behavior and neural activity. Ann N Y Acad Sci 1060: 400–403. 16597792 10.1196/annals.1360.044

[53] Bengtsson SL, Ullen F, Ehrsson HH, Hashimoto T, Kito Tet al (2009) Listening to rhythms activates motor and premotor cortices. Cortex 45: 62–71. 19041965 10.1016/j.cortex.2008.07.002

[54] Calvo-Merino B, Glaser DE, Grezes J, Passingham RE, Haggard P (2005) Action observation and acquired motor skills: an FMRI study with expert dancers. Cerebral Cortex 15: 1243–1249. 15616133 10.1093/cercor/bhi007

[55] Blasing B, Calvo-Merino B, Cross ES, Jola C, Honisch J, et al. 2012. Neurocognitive control in dance perception and performance. Acta Psychologica 139: 300–308. 22305351 10.1016/j.actpsy.2011.12.005

[56] Haueisen J, Knosche TR (2001) Involuntary motor activity in pianists evoked by music perception. J Cogn Neurosci 13: 786–792. 11564322 10.1162/08989290152541449

[57] D’Ausilio A, Altenmuller E, Olivetti Belardinelli M, Lotze M (2006) Cross-modal plasticity of the motor cortex while listening to a rehearsed musical piece. The European Journal of Neuroscience 24: 955–958. 16930423 10.1111/j.1460-9568.2006.04960.x

[58] Lahav A, Boulanger A, Schlaug G, Saltzman E (2005) The power of listening: auditory-motor interactions in musical training. Ann N Y Acad Sci 1060: 189–194. 16597764 10.1196/annals.1360.042

[59] Bangert M, Altenmuller EO (2003) Mapping perception to action in piano practice: a longitudinal DC-EEG study. BMC Neuroscience 4: 26. 14575529 10.1186/1471-2202-4-26 PMC270043

[60] Chen JL, Penhune VB, Zatorre RJ. 2008. Listening to musical rhythms recruits motor regions of the brain. Cerebral cortex. 18: 2844–2854. 10.1093/cercor/bhn042 18388350

[61] Chen JL, Penhune VB, Zatorre RJ. 2008. Moving on time: brain network for auditory-motor synchronization is modulated by rhythm complexity and musical training. J Cogn Neurosci. 20: 226–239. 10.1162/jocn.2008.20018 18275331

[62] Ohyama T, Nores WL, Murphy M, Mauk MD (2003) What the cerebellum computes. Trends in Neurosciences 26: 222–227. 12689774 10.1016/S0166-2236(03)00054-7

[63] Bastian AJ. 2006. Learning to predict the future: the cerebellum adapts feedforward movement control. Current Opinion in Neurobiology 16: 645–649. 17071073 10.1016/j.conb.2006.08.016

[64] Garcia-Casares N, Berthier Torres ML, Froudist Walsh S, Gonzalez-Santos P. (2011) Model of music cognition and amusia. Neurologia. 10.1016/j.nrl.2011.04.010 21658819

[65] Hutchinson S, Lee LH, Gaab N, Schlaug G. 2003. Cerebellar volume of musicians. Cerebral cortex. 13: 943–949. 10.1093/cercor/13.9.943 12902393

[66] Zatorre RJ, Chen JL, Penhune VB (2007) When the brain plays music: auditory-motor interactions in music perception and production. Nature Reviews Neuroscience 8: 547–558. 17585307 10.1038/nrn2152

[67] Sevdalis V, Keller PE (2011) Captured by motion: dance, action understanding, and social cognition. Brain Cogn 77: 231–236. 21880410 10.1016/j.bandc.2011.08.005

[68] Beste C, Schuttke A, Pfleiderer B, Saft C (2011) Music perception and movement deterioration in Huntington’s disease. PLoS Currents. 3: RRN1252. 10.1371/currents.RRN1252 PMC3176446 21938274

[69] Voyer D, Voyer S (1995) Bryden, M P (1995) Magnitude of sex differences in spatial abilities: A meta-analysis and consideration of critical variables. Psychological Bulletin 117: 250–270. 7724690 10.1037/0033-2909.117.2.250

[70] Jordan K, Wüstenberg T, Heinze HJ, Peters M, Jäncke L (2002) Women and men exhibit different cortical activation patterns during mental rotation tasks. Neuropsychologia 40: 2397–408. 12417468 10.1016/s0028-3932(02)00076-3

[71] Jansen-Osmann P, Heil M (2007) Suitable stimuli to obtain (no) gender differences in the speed of cognitive processes involved in mental rotation. Brain Cogn 64: 217–2728. 17433514 10.1016/j.bandc.2007.03.002

[72] Mohr C, Blanke O, Brugger P (2006) Perceptual aberrations impair mental own-body transformations. Behavioral Neuroscience 120: 528–34. 16768604 10.1037/0735-7044.120.3.528

[73] Seurinck R, Vingerhoets G, de Lange FP, Achten E (2004) Does egocentric mental rotation elicit sex differences? Neuroimage 23: 1440–9. 15589108 10.1016/j.neuroimage.2004.08.010

